# Subjective utility moderates bidirectional effects of conflicting motivations on pain perception

**DOI:** 10.1038/s41598-017-08454-4

**Published:** 2017-08-10

**Authors:** Susanne Becker, Wiebke Gandhi, Yan Jun Chen, Petra Schweinhardt

**Affiliations:** 1 Alan Edwards Centre for Research on Pain, McGill University, Montreal, Quebec H3A 0C7 Canada; 2 Faculty of Dentistry, McGill University, Montreal, Quebec H3A 0C7 Canada; 3 Department of Cognitive and Clinical Neuroscience, Central Institute of Mental Health, Medical Faculty Mannheim, Heidelberg University, Square J5, 68159 Mannheim, Germany; 4 Centre for Integrative Neuroscience & Neurodynamics, School of Psychology and Clinical Language Science, University of Reading, Reading, RG6 7BE UK; 5 Department of Neurology and Neurosurgery, Faculty of Medicine, McGill University, Montreal, Quebec H3A 0C7 Canada; 6 Interdisciplinary Spinal Research, Department of Chiropractic Medicine, Balgrist University Hospital, 8008 Zurich, Switzerland

## Abstract

Minimizing pain and maximizing pleasure are conflicting motivations when pain and reward co-occur. Decisions to prioritize reward consumption or pain avoidance are assumed to lead to pain inhibition or facilitation, respectively. Such decisions are a function of the subjective utility of the stimuli involved, i.e. the relative value assigned to the stimuli to compare the potential outcomes of a decision. To test perceptual pain modulation by varying degrees of motivational conflicts and the role of subjective utility, we implemented a task in which healthy volunteers had to decide between accepting a reward at the cost of receiving a nociceptive electrocutaneous stimulus or rejecting both. Subjective utility of the stimuli was assessed by a matching task between the stimuli. Accepting reward coupled to a nociceptive stimulus resulted in decreased perceived intensity, while rejecting the reward to avoid pain resulted in increased perceived intensity, but in both cases only if a high motivational conflict was present. Subjective utility of the stimuli involved moderated these bidirectional perceptual effects: the more a person valued money over pain, the more perceived intensity increased or decreased. These findings demonstrate pain modulation when pain and reward are simultaneously present and highlight the importance of subjective utility for such modulation.

## Introduction

“No pain, no gain” – this popular motto of athletes portrays in a nutshell well-known pain-pleasure dilemmas: prioritizing pleasure over pain can enhance pleasure. For example, enduring pain during a sports match can increase the pleasure of winning. When two opposed motivators such as pain and reward co-occur a conflict arises. The urges to minimize pain and to maximize pleasure become competitive desires that have to be evaluated simultaneously and weighted against each other. Enabling such evaluations, a hedonic continuum with pleasure and displeasure at its ends has been proposed^1, 2^.

The motivation-decision model of pain focuses on situations with motivational conflicts induced by co-occurring pain and reward, describing behavioral and perceptual consequences and possible neurobiological underpinnings^3, 4^. Anything that is evaluated as more important than pain in a specific situation should have antinociceptive effects. In contrast, if the pain is viewed as more important, pronociceptive effects should occur. These effects are presumed to be exerted via engagement or inhibition of descending opioidergic pathways, respectively^4^. In line with the model, reward-induced pain inhibition has been demonstrated in animal^5^ and human studies^6^, with the latter results suggesting that pain inhibition by reward increases with increasing motivational conflict.

Similar to receiving reward, avoiding aversive outcomes is desirable and pleasurable. Thus, according to the motivation-decision model, the decision to avoid an aversive outcome other than pain, e.g. a monetary loss, should also have pain-inhibitory effects. In turn, the decision to accept an aversive outcome in order to escape or avoid pain should have pain-facilitatory effects. While the effects of choosing between two negative outcomes have been tested^7^, conflicts induced by having to accept pain to avoid another negative outcome have not been investigated before.

Another aspect that has been neglected in the literature on the interaction of pain and reward/pleasure is how a motivational conflict arises. Such a conflict is determined by the subjective utility of the stimuli involved. Subjective utility is the value a person subjectively assigns to specific stimuli to compare the potential outcomes of a decision (cf.^8, 9^). The subjective utility of a stimulus depends on external and internal factors such as bodily needs, personal preferences, previous learning history, environmental conditions etc. For example, warmth is useful in a hypothermic state and therefore feels pleasant, but undesirable, and thus subjectively unpleasant, in a hyperthermic state^10^; food is satisfying in a hungry but not in a satiated state^11^. Thus, whether an organism decides to accept pain to get a reward or to avoid pain at the cost of not getting the reward is highly dependent on state variables. In addition, it can be expected that subjective utility depends on trait variables, e.g. how pain sensitive someone is. Subjective utility is known to cause large variations in human perception^9, 12^, but have been neglected in studies on the interaction of pain and reward/pleasure.

Given the gaps in the literature, the aims of this study were to (1) investigate facilitatory and inhibitory effects on perceived pain intensity with varying degrees of motivational conflicts induced by co-occurring pain and reward, either conceptualized by monetary wins or by the avoidance of monetary losses; (2) test modulatory effects of the subjective utility of pain avoidance and reward on such pain modulation.

## Material and Methods

### Participants

The sample size was calculated a priori and powered to detect a medium effect size (f = 0.25) with a 5% probability for committing a Type I error (α = 0.05) and a 20% probability for a Type II error (1-β = 0.80). Validated power and sample size methods exist only for limited classes of mixed models^13^ not adequate for the model used here. Therefore, the needed sample size was estimated for a repeated measures design with one group and 5 conditions (number of measurements) (G * Power 3)^14^, possibly overestimating the number of needed participants. This resulted in 21 needed participants (with a critical F = 2.49 and an actual power of 1-β = 0.82). Twenty-one healthy volunteers (11 female, 10 male; age *M* = 23.5 yrs, *SD* = 5.9 yrs, range 18–45 yrs) participated in this study, which is part of a larger double-blind, placebo-controlled, cross-over study using the dopaminergic D2-receptor antagonist sulpiride. Only data from the placebo session are presented here. Exclusion criteria were any present or past pain condition, psychiatric disorders, including pathological gambling, substance abuse behaviors, alcohol consumption of more than 100 ml alcohol per week, tobacco use, regular night shifts or sleep disorders. The study was approved by the McGill University Institutional Review Board and informed consent was obtained from all participants according to the revised Declaration of Helsinki (2013).

### Electrocutaneous stimulation

While participants were performing a decision-making task (see below) they received transcutaneous electrical stimuli using a constant voltage isolated linear stimulator (STMISOLA, BIOPAC Systems, Inc.). Stimuli were applied to the participants’ non-dominant volar forearm using disposable surface electrodes. Participants’ sensitivity to the electrocutaneous stimuli was assessed and stimulus intensities in the decision-making task individually adjusted to induce a strongly painful and/or unpleasant sensation (see below). Each stimulus in the task lasted two seconds, consisting of 100 two-millisecond pulses with 18 milliseconds inter-pulse intervals.

### Rating scales

Participants rated their sensation of the electrocutaneous stimuli using a horizontally orientated Visual Analogue Scale (VAS). We used a VAS combining intensity and unpleasantness assessment because pilot studies showed that some participants perceived the nociceptive stimuli as unpleasant but not clearly painful or vice versa, even if intensities were close to individual tolerance. Such dissonance between pain and unpleasantness ratings can most likely be attributed to the unnatural sensation induced by electrocutaneous stimuli. Therefore, to avoid distorted ratings, we used the combined VAS, acknowledging that this does decrease potentially the specificity of any results. The VAS ranged from 0 “no sensation” to 100 “extremely unpleasant or painful”. In addition, participants rated the hedonic experience (‘liking’) of the outcome of the task on another VAS, ranging from −100 “dislike very much” to 100 “like very much”. Participants were instructed to rate their liking of the monetary outcomes irrespective of the nociceptive stimuli in trials in which the two types of stimuli co-occurred. Before commencing with testing, participants were familiarized with the rating scales.

### Assessment of sensitivity to electrocutaneous stimulation

Participants’ sensitivity to the electrocutaneous stimulation was assessed prior to performing the decision-making task in a three-step procedure:

1. Participants were familiarized with the sensation and the range of intensities of the electrocutaneous stimuli. For this purpose, participants received 5–10 stimuli, starting with an intensity of 1.0 mA, increased by steps of 0.5 mA until they reported a rating of 90 or higher on the VAS.
2. Participants received eight stimuli with intensities in randomized order. Intensities of these stimuli were determined using the lowest intensity (mA) rated higher than 0 (no sensation) from the familiarization as minimum and the highest intensity from the familiarization (Step 1) as maximum, with equidistant intensities within this range. Participants rated each stimulus on the combined intensity/unpleasantness VAS.
3. To take into account that monetary reward affects sensitivity, participants received two more sets of the same eight stimuli as in Step 2 in newly randomized orders. After each stimulus presentation, participants were asked if they would accept to receive the same stimulus again in order to receive a monetary reward (yes/no answer). With the first set of eight stimuli, the reward offered was 5 cents and with the second set 15 cents.

Based on the sensitivity assessment, individual stimulus intensities for the decision-making task were estimated using psychometric functions. The aim was to obtain robust estimators of the stimulus intensities needed in the decision-making task to induce a motivational conflict between the applied nociceptive stimuli and the offered reward. Psychometric functions were fitted to the VAS ratings of the stimuli applied in Step 2 and the decisions in Step 3 of the sensitivity assessment, modeling the relationship between ratings and decisions at different stimulus intensities. Based on pilot tests, the intensities estimated by these psychometric functions corresponding to a rating of 70 on the VAS (Step 2) and for a 40% chance of avoiding the stimulation (Step 3) were used and averaged. The resulting mean intensity (mA) was used as the individual’s stimulus intensity in the decision-making task, which aimed to be high enough to induce a strongly painful sensation, while leaving room for decreasing and increasing perceived intensity.

### Decision-Making Task

A decision-making task was used in which participants had to weight receiving or rejecting monetary wins or losses against receiving or avoiding painful/unpleasant nociceptive stimuli.

**Figure 1:**
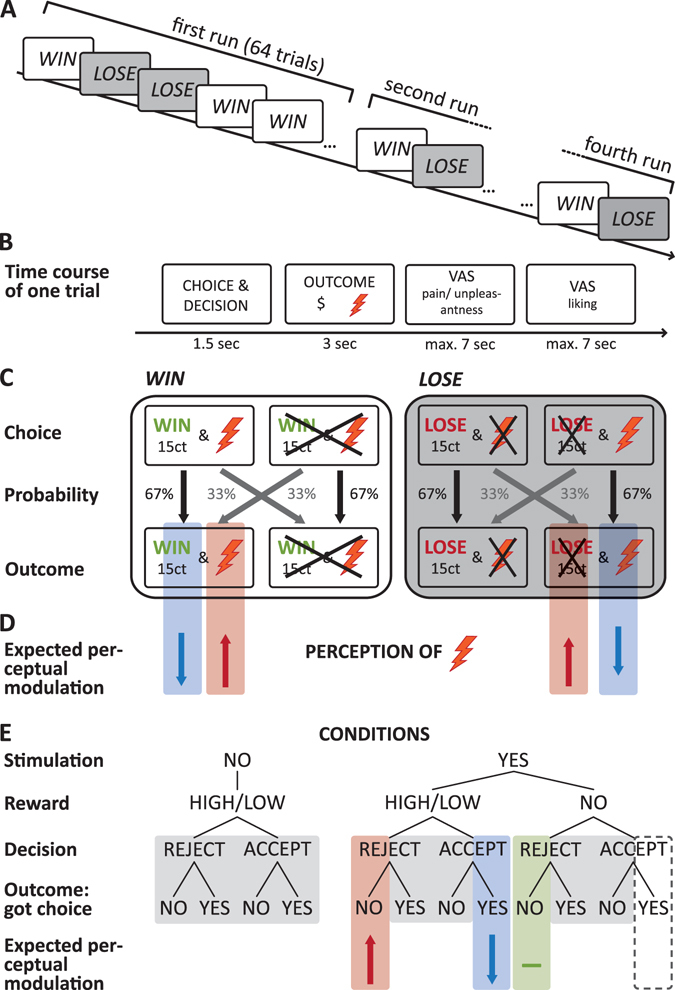
The decision-making task. (**A**) In four runs of 64 trials each, participants performed *win* and *lose* trials in pseudo-randomized order. (**B**) Time course of one trial: At the beginning of each trial the possible choices were displayed and participants indicated their decision, followed by the presentation of the outcome. If an electrocutaneous stimulus was applied, participants rated how painful and/or unpleasant they perceived this stimulus on a VAS. Subsequently, participants rated in each trials how much they liked the monetary outcome of the trials on a VAS. After a short break (1 sec) the next trial started. (**C**) In the *win* trials, participants had to decide whether they wanted to receive a monetary win at the cost of simultaneously receiving a nociceptive stimulus or to reject the win at the benefit of avoiding the pain. In the lose trials, participants had to choose between a monetary loss at the benefit of avoiding a nociceptive stimulus or rejecting the loss at the cost of receiving the stimulus. In both trial types, participants received their choice in 67% of the trials (outcome). (**D**) Decreased perceived intensity (blue shading) was expected if participants accepted the monetary win coupled to the nociceptive stimulus or when they rejected the loss coupled to the nociceptive stimulus. Increased perceived intensity (red shading) was expected if participants rejected the monetary win to avoid the nociceptive stimulus or when they accepted the loss to avoid the stimulus. (**E**) Coding of conditions for the statistical analyses: Trials with and without electrocutaneous stimulation could be coupled to low or high reward (in terms of receiving a monetary win or avoiding a monetary loss). Trials with stimulation could be also coupled to no (zero) reward. Participants could accept or reject the reward coupled to the electrocutaneous stimulation and they could get (67%) or not get (33%) their choice. Shaded areas indicate trials with no stimulation. Only for trials with stimulation effects on perceived pain could be analysed. Blue shading indicates the condition for which decreased perceived intensity was expected, red shading increased perceived intensity. Green shading indicates the control condition. The condition highlighted by a dotted line, indicates trials that were excluded from the main analyses because of low occurrences.

The task comprised two types of trials: the *win* trials, in which participants had the chance to win a certain amount of money and the *lose* trials, in which they had the chance to avoid the loss of a certain amount of money (Fig. 1A). Receiving monetary wins in the *win* trials and avoiding monetary losses in the *lose* trials operationalizes reward in both instances (positive reinforcement in *win* trials; negative reinforcement in *lose* trials). In the *win* trials, the following two options were displayed on a screen in front of the participants: accepting a monetary win (reward) coupled to simultaneous application of a painful/unpleasant electrocutaneous stimulus vs. rejecting the win and the electrocutaneous stimulus. In the *lose* trials, the two options were to accept a monetary loss at the benefit of avoiding a painful/unpleasant electrocutaneous stimulus vs. rejecting the monetary loss (reward) coupled to simultaneous application of the stimulus (Fig. 1C).

In both trial types, participants indicated their choice by pressing one of two keys on a keyboard. In 67% of cases participants received their choice, while in 33% they received the option they had not chosen (Fig. 1C). Whether participants received their choice or not was displayed immediately after participants made their choice. In trials with nociceptive stimulation, the electrocutaneous stimulus was applied while the outcome of the trial was displayed. To assess increases and decreases in perceived intensity, stimulus application was immediately followed by the previously described VAS on which participants rated their sensation of the stimulus (Fig. 1B).

It was expected that participants perceived the electrocutaneous stimulus as less intense or unpleasant, if they accepted a monetary reward (a monetary win or avoidance of a monetary loss) and as more intense or unpleasant if they rejected the reward to avoid the electrocutaneous stimulus (Fig. 1D). In order to test increased perceived intensity/unpleasantness when participants decided to reject the reward to avoid the nociceptive stimulus, it was necessary that participants did not receive their choice in a subset of trials (Fig. 1C).

The decision-making task comprised 256 trials in total, dived into four runs of 64 trials with short breaks between runs (Fig. 1A). 50% of the trials were *win* trials, 50% *lose* trials in an intermixed, predetermined pseudorandomized order. The number of applied electrocutaneous stimuli depended on participants’ choices.

Different amounts of monetary wins and losses binned into three categories were used: (1) *low*: wins/losses between 4–6 cents, mean 5 cents; (2) *high*: wins/losses between 11–15 cents, mean 13 cents; (3) *no*: zero wins/losses as a control condition. Categories of monetary wins or losses were applied either with or without nociceptive stimulation, except the combinations no win/loss with no stimulation, which were not used (Fig. 1E). Unbeknownst to the participants all applied electrocutaneous stimuli were of the same intensity.

Before participants started the decision-making task, they were familiarized with the different trial types and different options and the course of the task. During this familiarization, participants learned that in some instances they would not receive their choice, but were not informed about the percentage of trial in which they would not receive their choice. Participants were informed that they would receive their net win across all trials at the end of the session. To avoid a net loss, participants received 20$ to perform the task.

### Exit interview

At the end of the testing session an exit interview was performed to assess whether participants thought they received the placebo or the drug, what participants thought was the aim of the experiment, and whether participants perceive themselves as being more motivated to gain money or to avoid pain.

### Statistical analysis

#### Excluded data

For the analysis of the VAS ratings of the electrocutaneous stimuli, one extreme outlier (>3 standard deviations; rating of 0 in the condition high reward with pain) out of 1833 ratings was excluded from the statistical analyses. In addition, ratings of the electrocutaneous stimuli in the conditions with no monetary win or loss when participants accepted this zero win coupled to the electrocutaneous stimulus or accepted the electrocutaneous stimulus to avoid the zero loss were excluded from the main analyses because of very low occurrence (7 times for the wins and 20 for the losses). These trials were included in an analysis reported in the supplementary material (see S3) to compare effects of unexpected vs. expected electrocutaneous stimulation.

#### Analyses of VAS ratings

Normality of the VAS ratings was confirmed by skewness and kurtosis of the distributions (≤1). A repeated measurement ANOVA design using mixed model procedures was used to test the perceptual effects of accepting or rejecting rewards (in terms of receiving a monetary win or avoiding a monetary loss) coupled to a nociceptive stimulus. Specifically, VAS ratings of the nociceptive stimuli and liking ratings of the monetary outcomes served as dependent variables and the within-subject fixed factors ‘condition’ (see below for details on the coding of this factor) and ‘trial’ as a repeated independent factors. ‘Trial’ was included as a repeated factor nested within the factor ‘condition’ to account for possible effects of time over the course of the experiment. ‘Trial’ was included as a factor of no interest. If included as a factor of interest, the high number of trials nested within ‘Condition’ would create an unacceptably high number of parameters, preventing model convergence.

The different experimental manipulations and conditions, comprising (1) the category monetary wins or losses (low, high, no), (2) electrocutaneous stimulation (no stimulation, stimulation), (3) participant’s choice (accept, reject), (4) outcome of the trials (participants got their choice, did not get their choice) were jointly coded as the factor ‘condition’. This joined coding incorporated the fact some experimental conditions always co-occurred (see Fig. 1E). For example, for the analysis of the pain ratings, only trials with electrocutaneous stimulation could be analyzed. For these trials with stimulation, the options “reject” and “did not get choice” were always co-occurring as well as the options “accept” and “got choice”. By combining these options within one condition factor, redundancies and overfitting of the model was avoided.

The condition in which participants rejected 0$ as a “win” coupled to the electrocutaneous stimulus, but did not get their choice was used as the control condition in the win trials. In the lose trials, the condition in which participants accepted 0$ as a “loss” to avoid the electrocutaneous stimulus, but did not get their choice was used as the control condition (Fig. 1E).

*Win* and *lose* trials were analysed within separate statistical models. We did not aim to make direct comparisons between the two types of trials, because learning theory and findings on relief learning suggest that positive and negative reinforcement have different underlying mechanisms. Mixed model procedures were followed by post-hoc contrast calculations, when appropriate.

#### Analyses of subjective utility

To analyze whether subjective utility of the stimuli moderated perceptual effects and choice behavior in the decision-making task, an index of the integrated subjective utility of the electrocutaneous stimulation and the monetary reward was calculated. The subjective utility index was based on the lowest applied stimulation intensity participants rejected to receive 5 or 15 cents in Step 3 of the sensitivity assessment. Participants were ranked according to their index and the ranks from the assessment set using 5 cents and from the set using 15 cents were averaged for each participant. Higher values of the subjective utility index indicate that a participant was more motivated by the money in that she/he was willing to accept higher pain in order to obtain a reward. In turn, lower values indicate that a participant was more driven by the nociceptive stimuli.

The utility index was used in a moderator analysis implemented by a linear regression of the ratings of the nociceptive stimuli, including the main effect of the index and interaction with the factor ‘condition’, coded as above. Likewise, a log-linear regression (because of the categorical outcome variable) of participants’ choice behavior was calculated. The main effects of these regressions show whether subjective utility explains a significant amount of variance of the ratings or choice behavior and the interaction whether the utility moderates the perceptual effects and choice behavior across conditions. Moderator analyses were carried out separately for the *win* and *lose* trials.

#### Reporting of results

All results are reported in terms of Cohen’s d as effect sizes and interpreted as follows: d = 0.2 as a small, d = 0.5 as a medium effect, and d = 0.8 as a large effect^15^. To obtain consistent and comparable effect sizes independent of the used analysis approach, effect sizes were expressed in terms of Cohens’ d by transforming F-, z-, and r-values^15^. By this procedure, effects sizes are estimated and interpreted independent of sample size and the degrees of freedom, which might have led to an overestimation of the effects due to the high number of degrees of freedom in most analyses. For completeness, p-values are reported with a significance level of 5%.

Statistical analyses were performed using SPSS Statistics 21 (SPSS Inc. Chicago, USA) and RStudio 1.0.44 (RStudio, Inc.) with R 3.3.2 (The R Foundation for Statistical Computing).

### Data availability

The datasets generated and analysed during the current study are available from the corresponding author on reasonable request.

## Results

### Choice behavior

**Figure 2:**
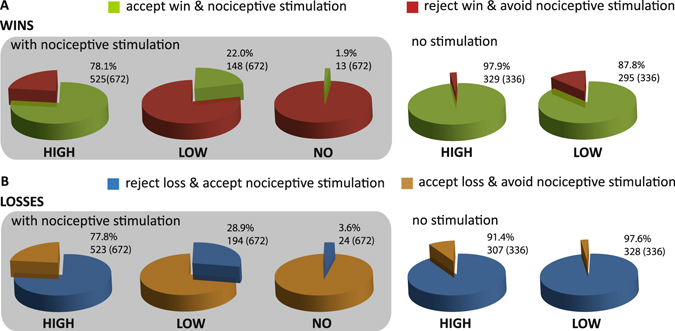
Choice behavior. (**A**) Percentage and absolute (out of) number of trials of accepted (green) vs. rejected (red) monetary wins coupled to a nociceptive stimulus (*win* trials). (**B**) Percentage and absolute (out of) number of trials of accepted (blue) vs. rejected (yellow) monetary losses coupled to pain avoidance (*lose* trials).

As intended, participants accepted in 22.0% of the *win* trials the low and in 78.1% the high reward coupled to the application of the electrocutaneous stimulus. Similarly, in the *lose* trials participants rejected in 28.9% the low and in 77.8% the high loss coupled to application of the electrocutaneous stimulus (see Fig. 2 for absolute numbers and the distribution of choices in the control condition and conditions of no interest).

### Perceptual effects of accepting or rejecting monetary wins coupled to an electrocutaneous stimulus (win trials)

**Figure 3:**
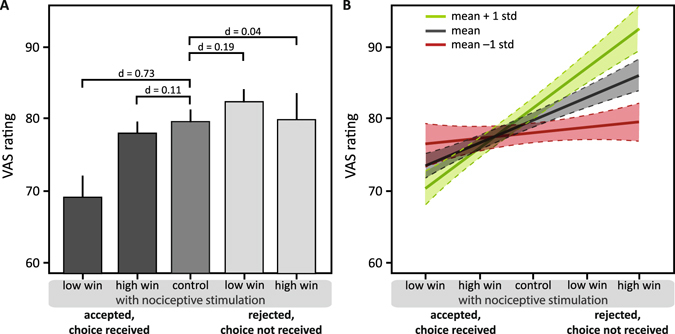
Perception of the nociceptive stimuli in *win* trials. (**A**) Means and 95% confidence intervals of VAS ratings in the five conditions with nociceptive stimulation: accepted and received with low win, accepted and received with high win, control condition, rejected and not received with low win, rejected and not received with high win. Post-hoc comparisons: effect size indicated by Cohen’s d. (**B**) Effects of subjective utility as a moderator are depicted by regression lines with 95% confidence intervals of estimated VAS ratings at below average (mean −1 standard deviation; green), average (mean; grey), and above average (mean +1 standard deviation; red) values of the utility index. The higher the values of the utility index, the higher the participant’s tendency to accept a win and a nociceptive stimulus and the stronger the perceptual modulation.

Perception of the electrocutaneous stimulus depended on whether participants accepted and received a monetary win coupled to the electrocutaneous stimulus or whether they rejected this win and the electrocutaneous stimulus but nevertheless received it (main effect ‘condition’ d = 0.29, F_876_ = 4.608, p = 0.001). Accepting a low win coupled to the electrocutaneous stimulus resulted in decreased perceived intensity compared to the control condition (post-hoc linear contrast d = 0.73, t_877_ = −2.76, p = 0.006; Fig. 3A), while accepting a high win had no effect on the perception of the electrocutaneous stimulus compared to the control condition (post-hoc linear contrast d = 0.11, t_874_ = −2.87, p = 0.004; Fig. 3A). Rejecting but nevertheless receiving a low monetary win resulted in a small increasing effect on perceived intensity of the electrocutaneous stimulus compared to the control condition (post-hoc linear contrast d = 0.19, t_873_ = 0.57, p = 0.528; Fig. 3A), while receiving a rejected high win had no effect on the perception of the electrocutaneous stimuli compared to the control condition (post-hoc linear contrast d = 0.04, t_877_ = 0.74, p = 0.385; Fig. 3A). (Please see supplementary materials S1 for results for low and high wins pooled).

### Modulatory role of preference to accept or avoid pain when coupled to a monetary win (win trials)

Whether participants had the tendency towards accepting an electrocutaneous stimulus to receive money or towards rejecting the money to avoid the stimulus (indicated by the utility index) moderated increasing or decreasing perceptual effects of accepting or rejecting and receiving the monetary win coupled to an electrocutaneous stimulus (main effect moderator d = 0.24, t_893_ = −3.58, p < 0.001; interaction of the moderator and ‘condition’ d = 0.36, t_893_ = 5.34, p < 0.001). The more participants were willing to accept an electrocutaneous stimulus to receive money, the greater the perceived intensity increase or decrease of the electrocutaneous stimuli in the *win* trials (Fig. 3B).

Condition effects of the moderator analysis showed that for individuals with below average values (mean – 1 std = 4.71) of the utility index, i.e. individuals who preferred to avoid pain at the cost of not getting a monetary win, the different conditions had no modulatory effect on the perception of the electrocutaneous stimulus (d = 0.07, t_893_ = 1.12, p = 0.264). In contrast, with average (mean = 6.02) and above average (mean + 1 std = 7.32) utility index values, the tendency of a participant to accept money albeit being coupled to receiving an electrocutaneous stimulus had a modulatory effect on the perception of the stimulus (mean rank: d = 0.45, t_893_ = 6.74, p < 0.001; mean + 1 std rank: d = 0.56, t_893_ = 8.43, p < 0.001), with stronger modulation the stronger the participant’s tendency to accept the win and the stimulus (Fig. 3B).

In contrast to the perception of the electrocutaneous stimulus, participants’ choice behavior was not moderated by their preference of accepting or avoiding a monetary win when coupled to an electrocutaneous stimulus (main effect moderator d = 0.04, z = −1.04, p = 0.30; interaction of the moderator and ‘condition’ d = 0.09, z = −2.33, p = 0.019).

### Perceptual effects of accepting or rejecting monetary losses coupled to the omission of an electrocutaneous stimulus (lose trials)

**Figure 4:**
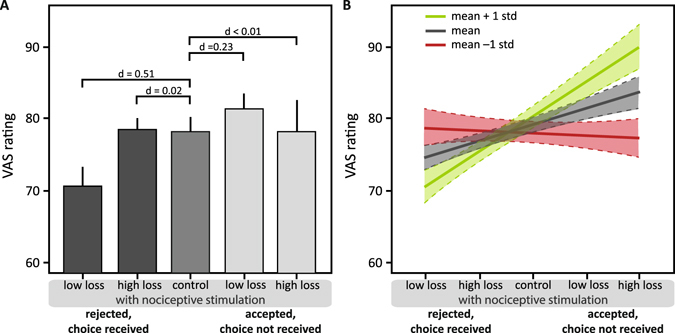
Perception of the nociceptive stimulation in *lose* trials. (**A**) Means and 95% confidence intervals of VAS ratings in the five conditions with nociceptive stimulation: rejected and received with low loss, rejected and received with high loss, control condition, accepted and not received with low loss, accepted and not received with high loss. Post-hoc comparisons: effect size indicated by Cohen’s d. (**B**) Effects of subjective utility as a moderator are depicted by regression lines with 95% confidence intervals of estimated VAS ratings at below average (mean −1 standard deviation; green), average (mean; grey), and above average (mean +1 standard deviation; red) values of the utility index. The higher the values of the utility index, the higher the participant’s tendency to accept a win and nociceptive stimulus and the stronger the perceptual modulation.

Similar to the effects in the *win* trials, only low losses affected the perception of the electrocutaneous stimuli: rejecting a low loss coupled to application of an electrocutaneous stimulus had a medium sized decreasing effect on perceived intensity of the electrocutaneous stimulus compared to the control condition (post-hoc linear contrast d = 0.51, t_885_ = −1.21, p = 0.23; Fig. 4A), while rejecting a high loss had no effect on the perception of the electrocutaneous stimulus (post-hoc linear contrast d = 0.02, t_882_ = 0.47, p = 0.64; Fig. 4A; main effect ‘condition’ d = 0.16, F_884_ = 1.47, p = 0.210). Accepting a low loss in order to avoid the electrocutaneous stimulus but nevertheless receiving both had a small increasing effect on perceived intensity of the electrocutaneous stimulus compared to the control condition (post-hoc linear contrast d = 0.23, t_882_ = 1.03, p = 0.32; post-hoc linear contrast: accepting a high loss to avoid the electrocutaneous stimulus but receiving both: d < 0.01, t_884_ = −0.99, p = 0.32; Fig. 4A). (Please see supplementary materials S2 for results for low and high wins pooled).

### Modulatory role of preference to accept or avoid pain when coupled to avoidance of monetary loss (lose trials)

Whether participants have the tendency toward accepting an electrocutaneous stimulus to receive money or toward avoiding the stimulus at the cost of not receiving the money (indicated by the utility index) moderated perceptual decreases and increased of accepting or rejecting and receiving the monetary loss coupled to an electrocutaneous stimulus (main effect moderator d = 0.34, t_902_ = −5.16, p < 0.001; interaction of the moderator and ‘condition’ d = 0.40, t_902_ = 6.01, p < 0.001). The more participants were willing to accept an electrocutaneous stimulus to receive money, the greater the perceived intensity increase or decrease of the electrocutaneous stimuli in the *lose* trials (Fig. 4B).

Effects of subjective utility as moderator in the *lose* trials were comparable to the *win* trials described above. Conditional effects of the moderator analysis showed that with below average (mean −1 std = 4.72) values in the utility index, indicating that for an individual who preferred to avoid pain at the cost of not getting a monetary win, the different conditions had no modulatory effect on perceived intensity of the electrocutaneous stimulus (d = 0.04, t_902_ = 0.59, p = 0.556). In contrast, with average (mean = 6.01) and above average (mean +1 std = 7.30) utility index values, the tendency of a person to accept money albeit being coupled to the reception of an electrocutaneous stimulus had a modulatory effect on perceived intensity of the stimulus (mean rank: d = 0.35, t_902_ = 5.19, p < 0.001; mean +1 std rank: d = 0.53, t_902_ = 7.89, p < 0.001), with stronger modulation the stronger participants’ tendency to accept the win and the stimulus (Fig. 4B).

As in the *win* trials, participants’ choice behavior was not moderated by their preference of accepting or avoiding a monetary win when coupled to an electrocutaneous stimulus (main effect moderator d = 0.03, z = 0.73, p = 0.46; interaction of the moderator and ‘condition’ d = 0.07, z = 1.79, p = 0.07, likelihood ratio test for interaction: χ^2^_1_ = 3.10, p = 0.08).

### Hedonic experience of the monetary outcomes

Ratings of the hedonic experience of the outcomes in the decision-making task differed between conditions in the *win* (main effect ‘condition’ d = 1.71, F_2341_ = 137.07, p < 0.001) as well as in the *lose* trials (main effect ‘condition’ d = 1.45, F_2464_ = 99.59, p < 0.001). However, ratings in the conditions without nociceptive stimulation were higher than the ratings in conditions with nociceptive stimulation within each reward condition (low, high), suggesting that participants’ hedonic ratings were influenced by receiving painful stimulation. Thus, conclusions on possible decreases or increases of the perception of the monetary outcomes in comparison to the control condition were not possible.

## Discussion

The present results show that accepting a reward at the cost of receiving a nociceptive stimulus decreased the perceived intensity of the stimulus. Conversely, rejecting such a reward in order to avoid the nociceptive stimulus increased the perceived intensity of the nociceptive stimulus. These perceptual decreases and increases are moderated by the subjective utility of the stimuli involved: the more a person values money over pain, the stronger were the bidirectional effects on perceived intensity. Such modulatory effects occurred regardless of whether reward was operationalized as positive reinforcement (receiving a positively valenced stimulus, here a monetary win) or negative reinforcement (avoiding a negatively valenced event, here a monetary loss).

The present results are in line with the predictions of Fields’ motivation decision model of pain^3, 4^: when participants prioritized receiving the reward over pain avoidance, pain-inhibitory effects occurred, while facilitatory effects occurred when participants prioritized pain avoidance over receiving the reward, demonstrated here on a perceptual level. To our knowledge, these results show for the first time increases of pain perception related to the decision to reject a reward at the expected benefit of pain avoidance, as predicted by the model. Earlier results demonstrated that the motivational conflict induced by co-occurring pain and monetary reward affects choice behavior and appears to attenuate pain avoidance behavior; however, perceptual effects were not assessed^16–18^. The occurrence of perceptual effects in this study depended on the presence of a motivational conflict in line with previous suggestions^6^. Perceptual modulation of the nociceptive stimulation occurred only in the conditions with low reward (low monetary win or avoidance of a low monetary loss), in which the decision to choose the reward at the cost of receiving the nociceptive stimulus was not obvious for the participants as indicated by their choice behavior (acceptance of this option only in 22% of cases in the *win* and 29% in the *lose* trials). In contrast, it appears that the conditions with high reward resulted in a lower motivational conflict: participants chose in the great majority of cases (78% in both *win* and *lose* trials) to accept the nociceptive stimulus to receive the reward. In line with the notion that the degree of motivational conflict determines pain modulatory effects, no decreases of perceived intensity were observed with high reward.

The modulatory effects of the perception of the nociceptive stimuli were moderated by how an individual weights pain and money against each other, i.e. the subjective utility of the stimuli. Although subjective utility modulates human perception^9, 12^, it has been neglected in studies on the interaction of pain and reward/pleasure. An exception are two studies testing predictions of different models on the integration of pain and reward into an overall subjective value and their neural correlates^19, 20^. However, these studies did not assess effects on pain perception and did not take into account between-subject variance due to variations in subjective utility.

Subjective utility and the perception of a motivational conflict induced by two stimuli are particularly important in real life. For example, an athlete for whom winning a certain match is very important will perceive pain from e.g. a sprained ankle differently compared to someone who cares not much about winning the match. In both situations, pain and a potential reward co-occur but perceptual effects are very different. In chronic pain, a motivational conflict might be more easily caused because pain and pain-related cues are more salient^21^, possibly more easily outweighing anticipated pleasure from a reward. If avoiding (increased) pain is prioritized over reward, pain facilitation occurs, as shown in the present study. At the same time, prioritizing pain over reward should lead to a devaluation of the reward^3, 4^. In line with this hypothesis, anhedonia, i.e. the inability to feel pleasure, has been described in chronic pain and impaired reward-dependent learning^22, 23^.

Results from animal research suggest that dopamine reward prediction error responses represent the integrated subjective value of co-occurring positive and negative stimuli^24, 25^. Dopaminergic functions are related via mesocortical pathways to the ventromedial prefrontal and orbitofrontal cortex (vmPFC/OFC)^26, 27^, regions in which the subjective value of different stimuli is integrated in terms of expected subjective value or utility^28^. Interestingly, both dopamine and the OFC have been shown to mediate pain-inhibitory effects when pain and reward are simultaneously present^29, 30^. Thus, possibly dopamine and the OFC might be the mediating mechanisms underlying the effects found here, but this has to be tested in future studies.

Receiving something positive and avoiding or escaping something negative induce both pleasure. While receiving something positive as a behavioral consequence is considered a positive reinforcement, avoiding or escaping a negative event is negative reinforcement, typically coupled to the perception of relief. Positive and negative reinforcement have been shown to differ regarding learned long-term effects^31^. But when coupled to a nociceptive stimulus, both types of reinforcement result in comparable effects on the perception of the nociceptive stimulus, as shown in the present study.

In order to test increases of perceived intensity when subjects rejected a monetary win or accepted a monetary loss in order to avoid a nociceptive stimulus, it was necessary to apply the nociceptive stimulus although participants had decided to avoid it. This results in violated expectations and could theoretically induce negative emotions such as frustration or disappointment increasing pain perception^32^. Comparing the control trials (no reward with electrocutaneous stimulation rejected but nevertheless received) with trials in which participants accepted trials with no (zero) win coupled to the electrocutaneous stimulus or trials in which participants accepted the electrocutaneous stimulus to avoid the no (zero) loss, showed no difference between trials in which participants received their choice compared to when not receiving their choice (see supplementary material, S3). Because of the low occurrence of the trials for comparison with the control trials (N = 7 in the *win* trials, N = 20 in the *lose* trials), these results have to be interpreted cautiously. Nevertheless, this finding, together with the pattern of perceptual modulation with low and high reward speaks against effects of negative emotions; effects of negative emotions would be expected to increase perception of the nociceptive stimuli with low and high rewards but increased perception was only observed with low reward (when participants had rejected this choice) for both the *win* and the *lose* trials. This indicates that it was indeed a pain modulatory effect of the decision to prioritize pain over reward that was observed.

For the hedonic ratings, participants were instructed to rate their liking of the monetary outcome independent of the pain. However, the hedonic ratings suggest that the nociceptive stimulus was too strong to be ignored. In contrast, the pain intensity and unpleasantness ratings suggest that participants truly only rated the nociceptive stimuli: if pain ratings had been influenced by how much participants liked the monetary wins, the ratings would have been lower when nociceptive stimulation was paired with high reward compared to being paired with low reward or no reward. However, pain ratings were comparable when nociceptive stimulation was paired with high reward and no reward. We are therefore confident that participants rated their perceptions of the nociceptive stimuli independent of the monetary outcomes. To avoid biased ratings because of dissociations between pain and unpleasantness sensations, we used a combined rating scale of pain intensity and unpleasantness. It should be noted that this combined rating scale does not allow separating sensory-discriminative and affective dimensions of pain, which are known to be mediated by different cortical systems^33^.

The context of the drug administration in this study could have influenced participants’ behavior in the testing session. However, because participants did not receive information regarding which sort of drug effects were expected, it is unlikely that specific expectations might have influenced pain ratings, in particular in any systematic way (i.e. in one experimental condition vs. another).

In sum, the present results highlight the importance of subjective utility of pain avoidance and (monetary) rewards in decreasing and increasing perceived pain intensity when participants decide to accept a monetary reward coupled to pain or reject a monetary reward to avoid pain. To further expand these findings, future studies could investigate whether dopamine and the OFC mediate the moderating role of subjective utility in modulation of the perception of nociceptive stimuli and whether these effects are altered in chronic pain patients.

## Electronic supplementary material

Supplementary Information
