# Supplementary material for: Subjective utility moderates bidirectional effects of conflicting motivations on pain perception
Source: Sci Rep. 2017 Aug 10;7:7790. doi: 10.1038/s41598-017-08454-4 (PMC5552734; doi:10.1038/s41598-017-08454-4)
Supplement: Supplementary file 1 — Supplementary Information [file 41598_2017_8454_MOESM1_ESM.pdf]

SUPPLEMENTARY MATERIAL

Subjective utility moderates bidirectional effects of conflicting motivations on pain perception

Susanne Becker[1,2,3]*, Wiebke Gandhi[1,2,4], Yan Jun Chen[1,2], Petra Schweinhardt[1,5,6]

[1] Alan Edwards Centre for Research on Pain, McGill University, Montreal, Quebec, H3A 0C7, Canada

[2] Faculty of Dentistry, McGill University, Montreal, Quebec, H3A 0C7, Canada

[3] Department of Cognitive and Clinical Neuroscience, Central Institute of Mental Health, Square J5, 68159 Mannheim, Germany

[4] Centre for Integrative Neuroscience & Neurodynamics, School of Psychology and Clinical Language Science, University of Reading, Reading, RG6 7BE, UK

[5] Department of Neurology and Neurosurgery, Faculty of Medicine, McGill University, Montreal, Quebec, H3A 0C7, Canada

[6] Interdisciplinary Spinal Research, Department of Chiropractic Medicine, Balgrist University Hospital, 8008 Zurich, Switzerland

S1. Perceptual effects of accepting or rejecting monetary wins coupled to an electrocutaneous stimulus (win trials) – low and high wins pooled

Independent of the amount of monetary wins, accepting the wins (low and high wins pooled) resulted in a small sized effect decreasing perceived intensity/unpleasantness of the electrocutaneous stimuli compared to the control condition (post-hoc linear contrast d = 0.23, t_{877} = - 3.30, p = 0.001). In contrast, rejecting a win and the electrocutaneous stimulus (low and high wins pooled) did not change the perception of the electrocutaneous stimuli compared to the control condition if the stimulus and the win were nevertheless applied (post-hoc linear contrast, d=0.14, t_{876} = 0.84, p = 0.404).

S2. Perceptual effects of accepting or rejecting monetary losses coupled to the omission of an electrocutaneous stimulus (lose trials) – low and high losses pooled

When low and high losses were pooled, rejecting a monetary loss at the cost of receiving an electrocutaneous stimulus or accepting the loss in order to avoid the stimulus but nevertheless receiving both had no effect on the perception of the electrocutaneous stimulus compared to the control condition (main effect 'condition' d = 0.16, F_{884} = 1.47, p = 0.210; post-hoc linear contrast: losses accepted to avoid the stimulus: d = 0.13, t_{884} = -0.56, p = 0.573; post-hoc linear contrast: losses rejected and stimulus accepted: d = 0.17, t_{884} = -0.24, p = 0.812).

S3. Comparison of control trials and trials in which participants accepted a zero reward coupled to the electrocutaneous stimulus.

To compare whether ratings of perceived pain and/or unpleasantness differed when participants received the electrocutaneous stimulus after having it accepted compared to having it rejected, we compared for the *win* trials the trials in which participants accepted a zero win coupled to the electrocutaneous stimulus (N=7) and trials in which participants rejected a zero win coupled to the electrocutaneous stimulus but nevertheless received it (control trials) and for the *lose* trials the trials in which participants accepted the electrocutaneous stimulus to avoid the zero loss (N=20) and trials in which participants rejected a zero loss coupled to avoidance of the electrocutaneous stimulus but nevertheless received it (control trials) in separate mixed model ANOVA procedures with the within-subject fixed factors 'choice' (electrocutaneous stimulus accepted vs. rejected) and 'trial' as a repeated independent factors.

Perception of the electrocutaneous stimulus was not different when participants rejected the stimulus (coupled to a zero reward) but nevertheless received it compared to when they accepted to stimulus (coupled to a zero reward) neither in the *win* (main effect 'choice' d=0.06, F_{213} = 0.22, p = 0.64) nor in the *lose* trials (main effect 'choice' d=0.15, F_{182} = 1.07, p = 0.30).

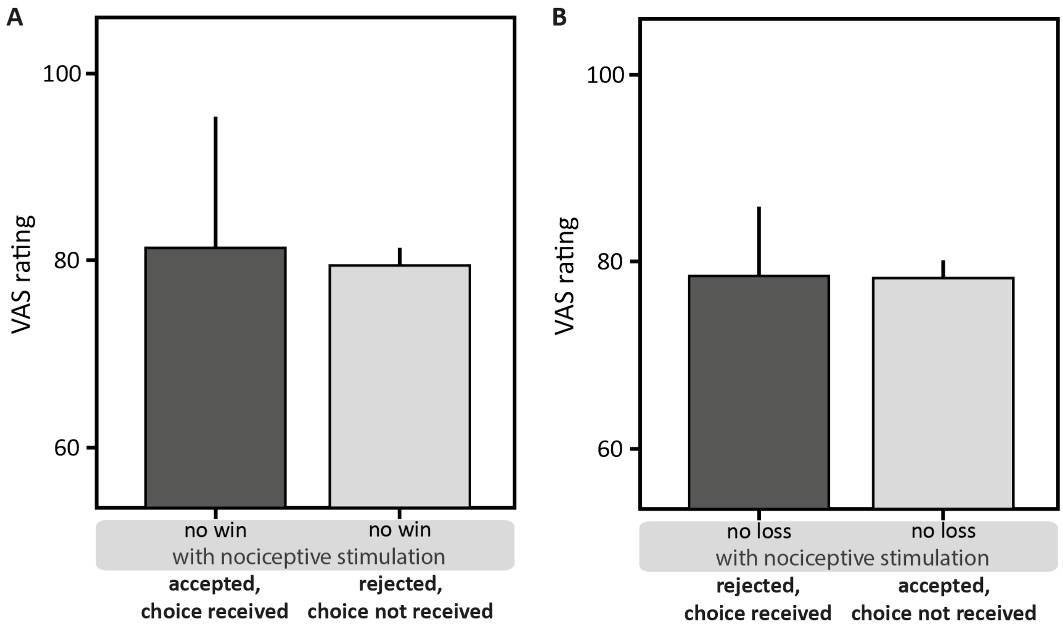

Figure S3. Perception of the electrocutaneous stimulation in trials with zero reward when participants accepted or rejected the stimulation. (A) *Win* trials: Means and 95% confidence intervals of VAS ratings in trials in which participants accepted a zero win coupled to the electrocutaneous stimulus (N=7) and trials in which participants rejected a zero win coupled to the electrocutaneous stimulus but nevertheless received it (control trials) (B) *Lose* trials: Means and 95% confidence intervals of VAS ratings in trials in which participants rejected the zero loss coupled to the electrocutaneous stimulus (N=20) and trials in which participants accepted a zero loss to avoid of the electrocutaneous stimulus but nevertheless received it (control trials).
